# Maternal adverse childhood experiences and their association with preterm birth: secondary analysis of data from universal health visiting

**DOI:** 10.1186/s12884-022-04454-z

**Published:** 2022-02-16

**Authors:** Katie Hardcastle, Kat Ford, Mark A. Bellis

**Affiliations:** 1 Policy and International Health, World Health Organization Collaborating Centre on Investment for Health and Well-being, Public Health Wales, Wrexham Technology Park, Wrexham, LL13 7YP UK; 2 Public Health Collaborating Unit, School of Medical and Health Sciences, Bangor University, Wrexham Technology Park, Wrexham, LL13 7YP UK

**Keywords:** Adverse childhood experiences, Child maltreatment, Childhood sexual abuse, Preterm birth, Maternal mental health

## Abstract

**Background:**

Being born before full gestation can have short-term and life-long health implications, yet it remains difficult to determine the risk of preterm birth among expectant mothers. Across different health settings, increasing attention is given to the health and behavioural consequences of adverse childhood experiences (ACEs) such as child abuse or neglect, or exposure to harmful household environments (e.g. in which caregivers abuse alcohol), and the potential value of understanding these hidden harms when supporting individuals and families. A large international evidence base describes the association between childhood adversity and early years outcomes for mothers and children. However, the relationship between maternal ACEs and preterm birth has received far less attention.

**Methods:**

Secondary analysis was carried out on anonymised cross-sectional data from health visiting services in south and west Wales that had previously captured information on mothers’ ACEs during routine contacts. Demographic data and information on mothers’ health were extracted from the Healthy Child Wales Programme.

**Results:**

Half of all mothers sampled had experienced at least one ACE, with a history of ACEs more common among younger, white British mothers and those residing in deprived areas. Preterm birth was significantly independently associated with retrospective reports of childhood sexual abuse (adjusted odds ratio [AOR] = 3.83, 95% confidence interval [CI] = 1.19–12.32, *p* = 0.025), neglect (AOR = 7.60, 95%CI = 1.81–31.97, *p* = 0.006) and overall ACE exposure (AOR = 2.67, 95%CI = 1.14–6.23, *p* = 0.024), with one in ten mothers (10.0%) who experienced ≥4 ACEs having preterm birth. Sub-analyses revealed a more pronounced relationship among mothers with no known chronic health conditions, with those with ≥4 ACEs and no known chronic condition four times more likely to give birth preterm (AOR = 3.89, 95%CI = 1.40–10.80, *p* = 0.009).

**Conclusions:**

Findings highlight the importance of the entire maternal experience. The experience of childhood adversity can have a lasting impact into and beyond the prenatal period, potentially increasing the risk of preterm birth, even among otherwise healthy women. Increasing our understanding of the potential perinatal outcomes associated with ACEs can help to inform how maternity services and partners offer trauma-sensitive support to mitigate some of the risks of early parturition, as well as target intergenerational cycles of adversity and poor health.

**Supplementary Information:**

The online version contains supplementary material available at 10.1186/s12884-022-04454-z.

## Background

In the UK, infants born before 37 weeks gestation are considered preterm or premature. Preterm birth may follow spontaneous onset of early labour or may be the result of an obstetric intervention intended to reduce the risk of continued pregnancy, to either the child and/or the mother [1]. According to the Office for National Statistics, 7.8% of live births were considered preterm in England and Wales in 2019 [2] and preterm birth accounts for 35% of all neonatal death globally [3]. Risk factors for preterm birth include demographic variables such as the extremes of maternal age [4] or low socio-economic status (SES) [5], health-harming behaviours and pre-existing health problems (e.g. maternal smoking [6] and obesity [7]), and pregnancy-related factors such as multiple gestation or intra-uterine infection [8]. However, in many instances, the cause of a preterm birth cannot be identified [9]. Birth before full gestation can have both short-term health implications, and negative impacts throughout the life course [10]. For example, the immune systems of preterm infants are immature, resulting in reduced innate and adaptive immunity [11]. Preterm birth is also known to increase the risk of a range of neurological disorders, including cerebral palsy, epilepsy and visual and cognitive impairment [12–14]. Whilst advances in perinatal care have significantly increased rates of survival [13], this often means families and healthcare services are left coping with the consequences of infants being born prematurely.

Increasingly, the potential impact of trauma and, in particular, historic or childhood experiences of chronic or toxic stress, on a range of perinatal outcomes has been considered [15–18]. Adverse childhood experiences (ACEs) is a collective term that refers to cumulative experiences of direct victimisation (e.g. physical or sexual abuse) or exposure to household dysfunction (e.g. a household member who abuses alcohol or is incarcerated) occurring during the first 18 years of life [19]. Established life course impacts of ACEs include increased risk of poor mental health, chronic health conditions such as type 2 diabetes and heart disease, and even early mortality [20–22]. Relationships between ACEs and subsequent development of health problems can be mediated by the presence or absence of resilience and other protective factors [23]. Although currently there is limited empirical evidence, plausible or theoretical links between ACEs and preterm birth have been suggested. These include both epigenetic and biological mechanisms [24], by which stress and inflammatory adaptive processes alter the brain, placenta and uterus, and thus the timing of parturition. Previous literature has examined the links between stress experienced in adulthood or during pregnancy and premature birth [25, 26]. However, recently studies have started to explore the role of childhood stress such as ACEs on preterm birth. Findings from a recent systematic review highlight that the majority of studies on this topic (*n* = 7/9 identified studies) suggest ACEs increase the risk for preterm birth [18]. However, no studies from the UK were identified in the review. A small prospective cohort study of African American women found that a history of childhood stress (in mothers) resulting from interpersonal loss and/or physical danger was associated with preterm birth, even when controlling for adult stress [27]. Although this relationship was postulated to be mediated by maternal cortisol, causal pathways remain unclear. Traumatic childhood experiences may not directly lead to increased risk for preterm birth, mediated instead by health behaviours. For example, studies have demonstrated links between ACEs, maternal alcohol consumption and preterm birth [28, 29].

Better understanding of the risk factors for preterm birth is an important element in both developing universal preventative measures, and in identifying those who are already at increased risk and may require additional support. Primary prevention interventions for preterm birth include nutritional supplementation and lifestyle and behaviour changes (e.g. smoking cessation, reducing occupational fatigue [30, 31]). Currently, despite advances in the use of biomarkers and ultrasound techniques (e.g. for cervical measurement [32]), when pregnancy begins, health practitioners have limited predictive ability for the relative risk of preterm birth [33], making it difficult to target treatment (e.g. prophylactic interventions), antenatal management decisions or support to reduce maternal and fetal risks. Many individual risk-scoring systems have not been subject to rigorous evaluation [34], and there are very few stress-related models to predict preterm birth [35]. Nevertheless, the likelihood is that a combination of tests may offer the best advances in clinical prediction [36].

Across a wide range of health and healthcare issues, new research and policy is now considering the application of ACE-informed approaches (e.g. [37]). In healthcare settings this includes health visiting (a universal service for families with children under the age of five freely provided through the UK National Health Service [NHS]) [38], in an attempt to better support those who have experienced ACEs and other childhood traumas and to break intergenerational cycles of harm and prevent offspring being exposed to adversity. At present, the ACE agenda is only beginning to be developed in an antenatal or midwifery context in the UK [39], and it is unclear if and how an understanding of historic childhood adversity may be relevant for health services in identifying and supporting vulnerable mothers. It is therefore important that we continue to increase our understanding of how ACEs may be associated with pregnancy outcomes.

## Methods

### Study aim, design, setting and data extraction

This study aims to explore the relationship between a history of ACEs and preterm birth among mothers engaged with health visiting services in south and west Wales. To our knowledge this is the first study to explore the associations between preterm birth and ACEs in a UK sample and furthers the evidence base on this topic through its examination of the role of maternal chronic health conditions on this relationship. Secondary analysis was carried out on anonymised cross-sectional data extracted from three health visiting services in urban and rural locations in south and west Wales in 2019/20. The study population is female primary caregivers (herein referred to as ‘mothers’) for whom ACE and Healthy Child Wales Programme (HCWP) data were made available by the health visiting services (see Measures). Health visitors completed data collection with mothers during their routine six-week post-partum contact on their retrospective ACE exposure (90.7% of those asked accepted to complete ACE enquiry). Information from the mothers’ HCWP record was also extracted. All data collection forms were subsequently stored in the mother’s paper file. Administrative assistants in each health visiting service ensured data collection forms contained no identifiable information (redacting if required) before scanning copies to the lead author using secure NHS sharing platforms. Data were entered manually by the research team into SPSS v24 for cleaning and statistical analysis. All data entry was quality assured. Cases which were missing data on key demographic variables including age, ethnicity and preterm birth (*n* = 49) were removed from the sample, leaving a final sample for analysis of 865.

### Measures

#### HCWP data – demographics and health

Data were extracted from the HCWP on mothers’ age, split into three discrete age categories (16–25; 26–35; ≥36 years). Owing to its protected category status under General Data Protection Regulation (GDPR), mothers were asked by their health visitor to indicate their ethnicity using only a dichotomous variable (‘white British’ or ‘Other’). In Wales, residential areas with higher deprivation are eligible for additional early years support services, in the form of Flying Start. Funding for Flying Start is allocated according to the estimated number of 0–3 year olds living in income benefit households in lower super output areas (LSOAs) of local authority areas. In 2018–19, over 36,000 children in Wales benefitted from Flying Start [40], which is used here as a proxy for deprivation. Mothers were also identified as living in Swansea, Carmarthenshire or Blaenau Gwent. HCWP data collection forms identified first-time mothers (first child: yes/no), preterm birth (i.e. birth before 37 completed weeks of gestation: yes/no) and provided data on relationship/marital status using the following categories: single (including separated, divorced or widowed); partnered/cohabiting; married; not disclosed. Further data were provided on mothers’ identified gender and sexual orientation, but were not included in analyses due to small numbers (< 5) within some categories.

As part of the 6 weeks post-partum contact, health visitors routinely complete the Family Resilience Assessment Instrument and Tool (FRAIT). The FRAIT uses a series of questions and scales to assist health visitors in making robust, consistent and reliable assessments of family resilience and in identifying the support and interventions needed to help families to deal with adversity [41]. The tool is split into five subscales, one of which (Family Health) is used to identify the mother’s chronic health conditions. The subscale is scored from one to five, with one representing parental chronic health problems that have a constant impact on the child’s needs, and five describing parents with no chronic health issues. For the purposes of analyses here, a FRAIT score of 1–4 was used to identify mothers with one or more chronic health condition(s).

#### Adverse childhood experiences (ACEs)

Health visiting services providing data for this secondary analysis were those that had previously used a simplified version of established ACE questions from the Centers for Disease Control and Prevention short ACE tool [42] to retrospectively measure caregivers’ childhood exposure (before they were 18 years of age) to the following ten ACE types: verbal abuse; physical abuse; sexual abuse; neglect; witnessing domestic violence; parental separation or divorce and parental/caregiver/household member mental illness; alcohol abuse; substance abuse; or incarceration. Mothers self-completed a written ACE questionnaire, alone or with the support of their health visitor if requested (e.g. when mothers had poor literacy), indicating yes/no to each ACE. For the purposes of analysis, mothers’ overall ACE exposure was categorized into 0, 1, 2–3 and ≥ 4 ACEs; as is consistent with methodologies applied elsewhere [19, 21]. These categories are intended to illustrate potential differences in outcomes by level of ACE exposure and current evidence does not identify them as having practical application for screening or intervention within health visiting services.

### Statistical analysis

Analyses used chi squared tests for initial bivariate examination of the relationships between ACEs and preterm birth. Binary logistic regression was also used to examine the independent contributions of ACEs and demographics (age; ethnicity; Flying Start; area; first child; relationship status) to these outcomes. Three separate models were run to explore the effects of individual ACE types (Model 1), total ACE exposure (Model 2) and all ACE measures together (Model 3). Generalized Linear Models (GLMs) allow covariate and categorical variables to be fitted to dependent variables and the resultant model can be used to generate estimates for the dependent variable for given values of the independent variable [43]. GLMs were used to generate adjusted means (i.e. estimated marginal means [EMMs], taking into account demographic differences between individuals; e.g. age, ethnicity and deprivation) for preterm birth for individuals with different levels of ACE exposure. Sub-analyses were also conducted to compare the modelled estimates of the impact of ACEs on preterm birth for mothers with and without chronic health conditions.

## Results

Over 60 % (64.3%) of mothers in the study population were aged 26–35 years, with just under a quarter (23.0%) aged 16–25 years. Married (31.0%) and partnered/cohabiting mothers (39.3%) made up the majority of the sample, although around one in every five (21.4%) mothers chose not to disclose their relationship status. Reflective of the overall ethnic diversity of the adult population of Wales [44], 90.8% of sampled mothers identified as white British. Over a quarter (27.9%) of mothers were in receipt of Flying Start services – representing the more disadvantaged areas of Wales. Overall, mothers had a mean of 1.8 children (SD = 0.97), with first time mothers accounting for just under half (46.8%) of the sample and only 2% of mothers having had a multiple birth (i.e. the birth of more than one infant from a single pregnancy). Of mothers in this sample, 4.7% had given birth (for the child[ren] they were currently receiving health visitor contact regarding) before 37 weeks gestation (preterm birth; Table 1).

**Table 1:** Associations between ACE exposure, demographics and preterm birth

	Total sample		Preterm birth
	N	%	%
All	865	–	4.7
Total ACE exposure			
0 ACEs	408	47.2	3.9
1 ACE	213	24.6	3.3
2–3 ACEs	134	15.5	5.2
≥ 4 ACEs	110	12.7	10.0
χ^2^			**8.412**
*p*			**0.038**
Age category (years)			
16–25	199	23.0	3.0
26–35	556	64.3	4.5
> 36	110	12.7	9.1
χ^2^			5.996
*p*			0.050
Ethnicity			
White British	785	90.8	5.1
Other	80	9.2	1.3
χ^2^			2.378
*p*			0.123
Pilot area			
Swansea	449	51.9	5.3
Carmarthenshire	224	25.9	4.0
Blaenau Gwent	192	22.2	4.2
χ^2^			0.763
*p*			0.683
Deprivation			
Non-Flying Start	624	72.1	4.3
Flying Start	241	27.9	5.8
χ^2^			0.846
*p*			0.358
First child			
No	460	53.2	4.8
Yes	405	46.8	4.7
χ^2^			0.004
*p*			0.950
Relationship status			
Single	72	8.3	6.9
Partnered/cohabiting	340	39.3	5.9
Married	268	31.0	3.7
Not disclosed	185	21.4	3.2
χ^2^			3.279
*p*			0.351

*ACE* Adverse childhood experience

### Maternal ACE exposure

Half of all mothers sampled (52.8%) reported that they had been exposed to one or more of the ten ACEs captured by the health visiting service, with 12.7% reporting high ACE exposure (≥4 ACEs; Table 1), in line with prevalence identified in national surveys [23]. Among this sample, ACE exposure differed significantly by age, with younger mothers (aged 16–25 years) reporting higher ACE exposure (17.6% ≥4 ACEs, compared with 11.9% of mothers aged 26–35 and 8.2% of those aged 36 years and over; χ^2^ = 21.073, *p* = 0.002). Reported experience of measured childhood adversity was also more common among white British mothers (compared with all other ethnicities; χ^2^ = 15.509, *p* = 0.001). Although no significant difference in ACE exposure was found by health visiting service/geographical area, mothers in Flying Start areas (i.e. from more disadvantaged areas in Wales) reported higher overall ACE exposure (≥4 ACEs; 17.0%, compared with 11.1% of non-Flying Start; χ^2^ = 11.610, *p* = 0.009) and a greater prevalence of the individual ACE types: physical abuse (14.9% vs 8.5%; χ^2^ = 7.822, *p* = 0.005), witnessing domestic violence (18.7% vs 13.0%; χ^2^ = 4.525, *p* = 0.033), household mental illness (26.1% vs 19.1%; χ^2^ = 5.232, *p* = 0.022), household alcohol abuse (19.1% vs 12.5%; χ^2^ = 6.143, *p* = 0.013), household drug abuse (7.9% vs 4.5%; χ^2^ = 3.904, *p* = 0.048) and household member incarceration (8.7% vs 2.1%; χ^2^ = 20.188, *p* < 0.001).

### Individual ACEs and preterm birth

Bivariate analyses revealed a significant positive association between mothers’ historic experiences of all measured forms of child maltreatment (verbal, physical, sexual abuse and neglect) and subsequent preterm birth (see Additional Table A1). Further, prevalence of preterm birth was significantly higher among mothers who had grown up witnessing domestic violence in their childhood home, and those who lived with parents or caregivers that experienced mental health issues (see Additional Table A1). When controlling for socio-demographic characteristics in multivariate analyses, significant independent effects on preterm birth remained for experiences of childhood sexual abuse and neglect (Table 2; Model 1).

**Table 2:** Logistic regression of individual and total ACE exposure, demographics and their association with preterm birth

	Model 1				Model 2				Model 3			
	AOR	Low CI	High CI	***p***	AOR	Low CI	High CI	***p***	AOR	Low CI	High CI	***p***
**ACE types**												
Verbal abuse	1.10	0.27	3.74	0.989					1.57	0.34	7.29	0.566
Physical abuse	0.88	0.24	3.25	0.849					0.94	0.25	4.02	0.994
Sexual abuse	**3.83**	**1.19**	**12.32**	**0.025**					**5.43**	**1.45**	**20.34**	**0.012**
Neglect	**7.60**	**1.81**	**31.97**	**0.006**					**9.12**	**1.92**	**43.37**	**0.005**
Parental separation	0.75	0.35	1.60	0.456					1.36	0.40	4.52	0.642
Domestic violence	1.78	0.63	4.98	0.276					2.78	0.76	10.14	0.122
Mental health	1.41	0.62	3.23	0.416					2.27	0.70	7.29	0.170
Alcohol abuse	1.15	0.42	3.17	0.787					1.53	0.48	4.92	0.474
Drug abuse	0.40	0.08	2.03	0.269					0.39	0.07	2.14	0.278
Incarceration	1.23	0.26	5.84	0.793					1.51	0.29	7.80	0.622
**Total ACE exposure**												
0 ACEs					(ref)			0.073	(ref)			0.630
1 ACE					0.80	0.32	2.00	0.635	0.50	0.14	1.86	0.303
2–3 ACEs					1.17	0.46	2.97	0.740	0.31	0.04	2.55	0.278
≥ 4 ACEs					**2.67**	**1.14**	**6.23**	**0.024**	0.10	0.00	3.38	0.201
**Age category (years)**												
16–25	(ref)			0.009	(ref)			0.011	(ref)			0.008
26–35	1.94	0.72	5.21	0.190	2.21	0.85	5.74	0.103	1.96	0.72	5.33	0.187
> 36	**5.48**	**1.72**	**17.50**	**0.004**	**5.48**	**1.76**	**17.06**	**0.003**	**5.69**	**1.76**	**18.41**	**0.004**
**Ethnicity**												
Other	0.17	0.02	5.21	0.098	0.22	0.03	1.66	0.142	0.19	0.02	1.54	0.121
**Pilot area**												
Swansea	(ref)			0.390	(ref)			0.476	(ref)			0.341
Carmarthenshire	0.61	0.25	1.39	0.237	0.63	0.28	1.41	0.261	0.57	0.25	1.32	0.190
Blaenau Gwent	0.64	0.26	1.55	0.321	0.72	0.31	1.68	0.450	0.64	0.26	1.55	0.324
**Deprivation**												
Flying Start	1.33	0.64	2.75	0.455	1.32	0.66	2.65	0.413	1.30	0.65	2.58	0.458
**First child**												
Yes	1.28	0.64	2.54	0.483	1.20	0.62	2.33	0.590	1.30	0.65	2.58	0.458
**Relationship status**												
Single	(ref)			0.593	(ref)			0.337	(ref)			0.595
Partnered/cohabiting	1.02	0.34	3.08	0.98	0.90	0.31	2.57	0.837	1.00	0.33	3.02	0.997
Married	0.67	0.19	2.38	0.54	0.51	0.16	1.70	0.275	0.67	0.19	2.34	0.527
Not disclosed	0.55	0.15	2.06	0.38	0.44	0.12	1.58	0.208	0.37	0.15	2.04	0.208

Model 1 = Individual ACEs, Model 2 = Total ACE exposure, Model 3 = Individual ACEs and total ACE exposure. Reference categories for dichotomous variables: white British ethnicity; non-Flying Start; has other children (first child = no)

*ACE* Adverse childhood experience, *AOR* Adjusted odds ratio, *CI* Confidence interval, *Ref* Reference category

Supplementary analyses revealed that all individual ACE measures were highly correlated in this sample, as is reported elsewhere [45, 46] (see Additional Table A2). Therefore, further analyses considered levels of overall ACE exposure as a potential predictor of preterm birth.

### Total ACE exposure and preterm birth

**Fig. 1:**
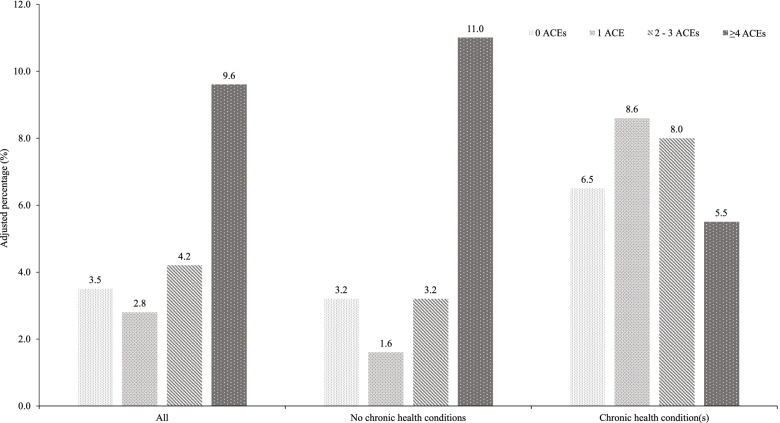
Modelled proportion of preterm birth by maternal ACE exposure and chronic health conditions*. *Adjusted for age, ethnicity and deprivation

Mothers’ overall exposure to adversity in childhood was significantly positively associated with preterm birth in bivariate analyses (Table 1). An independent effect of high ACE exposure remained when controlling for age and other socio-demographics in multivariate analyses, with mothers exposed to ≥4 ACEs over two and half times more likely than those with no ACEs to have preterm birth (Table 2; Model 2). No independent effect of ethnicity or deprivation on preterm birth was found. Modelled proportions (EMMs) of mothers who had a preterm birth ranged from 3.5% of those with no ACEs, to 9.6% among those experiencing high ACE exposure (≥4 ACEs) in the first 18 years of life (adjusted for age, ethnicity and deprivation; Fig. 1).

### Preterm birth and maternal chronic health problems

According to FRAIT data (see Methods), 15.7% of sampled mothers had one or more chronic health problem(s). Due to the strong theoretical and evidenced links between ACEs and maternal health, and maternal health and preterm birth [5, 8, 21], additional sub-analyses were conducted using ‘healthy’ mothers with no known chronic health conditions only (*n* = 709). For this sample, a more pronounced significant relationship between overall ACE exposure and likelihood of preterm birth was revealed, with mothers exposed to ≥4 ACEs almost four times more likely to have given birth preterm (AOR = 3.89; 95%CI = 1.40–10.80; *p* = 0.009; see Additional Table A3). The adjusted prevalence of preterm birth by ACE exposure for healthy mothers, those with chronic health conditions and the whole sample is shown in Fig. 1.

## Discussion

Being born before full gestation can have profound life course impacts. This study adds to a growing body of evidence which highlights the potential reproductive and perinatal impact of maternal childhood adversity [18]. In the first known exploratory analysis of its kind for mothers in the UK, evidence was found of an association between high ACE exposure and preterm birth, with almost one in ten mothers with ≥4 ACEs giving birth before 37 weeks gestation. As with studies reported elsewhere [45, 46], experiences of individual ACEs among these new mothers are inter-correlated. Therefore, whilst it is not clear if total ACE burden or specific ACEs are most influential, in support of previous research exploring the impact of historic sexual violence [9, 47–50], findings revealed that mothers’ experiences of childhood sexual abuse increased the likelihood of preterm birth almost four-fold. Existing evidence on the impact of the ACE type childhood neglect on reproductive and perinatal health is limited [51]. Nevertheless, mothers in this sample who reported experiences of neglect in childhood were actually over seven and a half times more likely to give birth prematurely, highlighting the importance of considering the totality of the maternal childhood experience. The strength of the relationship between ACEs and preterm birth among mothers without known chronic health conditions, as identified in this analysis, warrants particular attention, as it underlines the potential importance of maternal mental health and wellbeing, which may be hidden harms in parturition. Individuals with known health conditions will often have received treatment addressing symptoms and also, in some cases, underlying physical and mental issues relating to their condition. Such issues are also associated with greater exposure to ACEs [21]. Here, we found a stronger relationship between ACEs and preterm birth in individuals with no other reported health issues. This finding needs further study. However, it may be related to less general support and intervention being provided to those yet to be diagnosed with a health condition or diagnosed health conditions potentially related to ACE exposure confounding statistical relationships directly between ACEs and preterm birth.

Experiencing ACEs in childhood can have developmental and behavioural consequences, both of which may be linked with preterm birth. When chronic stress overcomes resilience, activation of inflammatory processes and neuroendocrine responses (e.g. cortisol and epinephrine) that would normally maintain homeostasis can have a negative effect on the body, resulting in increased risk of impaired health and disease processes, including preterm birth [24, 52]. Prolonged stress can lead to changes in the brain, uterus and placenta, impacting the mechanisms involved in preparation for, and timing of, parturition [24, 53]. Maltreatment in childhood is associated with structural deficits in brain structure, function and connectivity [54] - abnormalities that are related to a range of mental health sequelae, such as depression and anxiety [52] - prenatal maternal risk factors for preterm birth [55–57]. Disrupted development and neurocognitive deficits also increase vulnerability for social and behavioural difficulties [58]. Thus, ACEs and other chronic stressors are strongly associated with a range of behaviours - often construed as ‘coping mechanisms’ [59] - that are harmful to both mother and infant and may mediate the relationship between adversity and preterm birth, such as smoking [60] and alcohol use during pregnancy [61, 62].

In a population with such hidden harms, the value of understanding an individuals’ history of childhood adversity as a potential predictor for health impacts including preterm birth, and thus providing direction for targeted support or intervention, requires further consideration. In many health systems, midwifery services already routinely enquire about forms of victimization such as domestic violence and female genital mutilation [63, 64], with available evidence suggesting that practitioners perceive value in these processes [65, 66], although barriers to delivery remain [67]. Approaches to asking mothers about their experiences of childhood adversity have also been piloted in prenatal care in the US [68] and postnatally with health visiting services in the UK [38], where initial findings support both the feasibility and acceptability of such models, although impacts are less well established [69]. As ACEs occur in the context of care-giving relationships, these historic experiences may make it difficult for mothers to build and maintain trusting relationships with midwifery services [70], compounded by the relationship between early adversity and challenges such as being triggered by physical examination, fear of childbirth and delivery difficulties [47]. Therefore any model of enquiry for ACEs and associated childhood trauma should be developed and delivered in conjunction with wider principles of trauma-informed care and facilitate access to trauma-specific interventions (e.g. tailored labour planning) where required [71].

It is important to note that not all individual ACEs showed an independent relationship with preterm birth, and many mothers with a history of ACEs gave birth at full gestation, highlighting that the relationship between early adversity and preterm birth is by no means deterministic. However, approaches to identifying ACEs may have universal benefits when working with families in the early years, to break known intergenerational cycles of adversity and help to prevent exposure to ACEs and associated risk of preterm birth in subsequent generations. Such work has the potential to not only support families during the critical period of the first thousand days for bonding, attachment and building resilience [72, 73], but also reduce demands on healthcare, with the total annual cost of preterm birth to the public sector in England and Wales estimated at over £2.9 billion [74]. ACE-informed approaches delivered antenatally may add a new dimension where countries or regions are already forging an ACE awareness framework for practitioner training and screening (e.g. California [75]) and may have even greater application in countries where rates of preterm birth are higher and support for mothers and preterm infants is less readily available.

### Limitations

Findings from this exploratory analysis should be interpreted in light of the following limitations. Firstly, prevalence of preterm birth identified in this sample is lower than national figures derived from the same time period (7.8% [2]). As the sample only includes mothers who were actively engaged with a health visitor at 6 weeks post-partum, it may under-sample mothers whose infants have more complex care needs (i.e. were in special care baby units at 6-weeks post-partum), who experienced a still birth, or whose infant did not survive to 6 weeks post-partum, outcomes which may be more likely among preterm births. The present sample may also under-sample mothers who were identified by midwifery services as high risk and therefore may have health visitor contacts at different timings, meaning the opportunity to ask about ACEs did not arise. Secondly, the voluntary provision of self-reported ACE information may introduce a source of bias based on mothers’ willingness to report ACEs to their health visitor and have such information retained within their health record. Just under one in ten who were offered ACE screening declined to participate and it was not possible to identify or exclude any bias created by non-participation. As in previous national surveys of ACEs, the retrospective nature of self-reporting also introduces the potential of recall bias, particularly for experiences that may have been repressed. Currently, there is insufficient data to understand whether experiencing a preterm birth may have an impact on recall and reporting of ACEs. Limited detail was provided on maternal health and such data is collected through health visitor assessment and not determined in conjunction with the mother, thus, may not take account of all relevant aspects of maternal health, particularly those that are less visible, thus also introducing a potential source of bias. No data were provided by health visiting services on health-harming behaviours such as prenatal maternal diet and alcohol consumption, which have known relationships with preterm birth. Data were also not available on a number of other potentially important covariates, including age of menarche. We took into account deprivation using flying start eligibility. However, there may still be relationships with childhood SES which we could not control for and further studies should seek to include a better measure of SES. Finally, in line with other research [76], our analysis included both individual and cumulative measures of ACEs (see model 3). However, strong relationships between these measures should be acknowledged in the interpretation of these findings. In spite of these limitations, which are inherent in a small-scale exploratory analysis of secondary data, these initial novel findings are important for generating interest in the potential relevance of an ACEs framework in antenatal care and helping to frame future research questions in this space.

## Conclusions

The prevalence of ACEs retrospectively self-reported by mothers in this sample echoes that of adults in other primary health settings and national surveys, supporting the established premise that childhood adversity is not rare, and that experiences of different forms of victimisation or household dysfunction often co-occur. The widespread existence of ACEs in society highlights the need to consider different approaches to support those who have experienced childhood adversity across a variety of settings. With high ACE exposure associated with an increased risk of preterm birth as much as four fold among healthy mothers with no chronic health conditions, additional research is needed to explore this association in more detail, including the balance of influence for individual and cumulative ACEs on perinatal outcomes. Maternity and other services that engage with families during critical periods of child development may be uniquely placed, not only to offer trauma-sensitive support to deal with the impacts of ACEs and potentially mitigate some of the risks of early parturition, but also to help break cycles of adversity and health and social consequences for future generations.

## Supplementary Information

**Additional file 1: Table A1.** Bivariate association between individual ACEs and preterm birth.

**Additional file 2: Table A2.** Inter-correlations between individual ACEs.

**Additional file 3: Table A3.** Logistic regression of ACE exposure, demographics and their association with preterm birth among healthy* mothers.

## Data Availability

The data that support the findings of this study are available from the health visiting services in south and west Wales, but restrictions apply to the availability of these data, which were used under license for the current study, and so are not publicly available. Data are however available from the authors upon reasonable request and with permission of the health visiting services in south and west Wales.

## Abbreviations

ACE: Adverse childhood experience
AOR: Adjusted odds ratio
CI: Confidence interval
EMM: Estimated marginal means
FRAIT: Family Resilience Assessment Instrument and Tool
GDPR: General Data Protection Regulation
GLM: Generalized linear model
HCWP: Healthy Child Wales Programme
LSOA: Lower super output area
REF: Reference category
SES: Socioeconomic status
