# Supplementary material for: Maternal adverse childhood experiences and their association with preterm birth: secondary analysis of data from universal health visiting
Source: BMC Pregnancy Childbirth. 2022 Feb 16;22:129. doi: 10.1186/s12884-022-04454-z (PMC8848970; doi:10.1186/s12884-022-04454-z)
Supplement: Supplementary file 1 — Additional file 1: Table A1. Bivariate association between individual ACEs and preterm birth. [file 12884_2022_4454_MOESM1_ESM.docx]

**Table A1. Bivariate association between individual ACEs and preterm birth**

|  | **Preterm birth** | | |
| --- | --- | --- | --- |
| % | 5.1 | χ² | p |
| **Verbal abuse** | **8.9** | **6.090** | **0.014** |
| **Physical abuse** | **8.7** | **3.897** | **0.048** |
| **Sexual abuse** | **18.8** | **15.077** | **<0.001** |
| **Neglect** | **25.0** | **19.331** | **<0.001** |
| Parental separation | 5.0 | 0.248 | 0.618 |
| **Domestic violence** | **10.0** | **10.044** | **0.002** |
| **Mental health** | **7.9** | **6.022** | **0.014** |
| Alcohol abuse | 7.8 | 3.386 | 0.066 |
| Drug abuse | 6.3 | 0.310 | 0.578 |
| Incarceration | 8.8 | 1.426 | 0.232 |
