# Supplementary material for: Maternal adverse childhood experiences and their association with preterm birth: secondary analysis of data from universal health visiting
Source: BMC Pregnancy Childbirth. 2022 Feb 16;22:129. doi: 10.1186/s12884-022-04454-z (PMC8848970; doi:10.1186/s12884-022-04454-z)
Supplement: Supplementary file 2 — Additional file 2: Table A2. Inter-correlations between individual ACEs. [file 12884_2022_4454_MOESM2_ESM.docx]

**Table A2. Inter-correlations between individual ACEs**

| Pearson Correlation (n) | **Verbal abuse** | **Physical abuse** | **Sexual abuse** | **Neglect** | **Parental separation** | **Domestic violence** | **Mental illness** | **Alcohol abuse** | **Drug abuse** | **Incarceration** |
| --- | --- | --- | --- | --- | --- | --- | --- | --- | --- | --- |
| **Verbal abuse** | 1 | .683** | .290** | .279** | .266** | .548** | .353** | .441** | .298** | .277** |
|  | (865) | (865) | (864) | (864) | (864) | (865) | (865) | (865) | (865) | (864) |
| **Physical abuse** | .683** | 1 | .236** | .287** | .217** | .486** | .301** | .361** | .238** | .186** |
|  | (865) | (865) | (864) | (864) | (864) | (865) | (865) | (865) | (865) | (864) |
| **Sexual abuse** | .290** | .236** | 1 | .096** | .071* | .214** | .229** | .129** | .115** | .181** |
|  | (864) | (864) | (864) | (863) | (863) | (864) | (864) | (864) | (864) | (863) |
| **Neglect** | .279** | .287** | .096** | 1 | .114** | .296** | .193** | .231** | .277** | 0.051 |
|  | (864) | (864) | (863) | (864) | (863) | (864) | (864) | (864) | (864) | (863) |
| **Parental separation** | .266** | .217** | .071* | .114** | 1 | .331** | .291** | .306** | .182** | .169** |
|  | (864) | (864) | (863) | (863) | (864) | (864) | (864) | (864) | (864) | (863) |
| **Domestic violence** | .548** | .486** | .214** | .296** | .331** | 1 | .342** | .439** | .378** | .271** |
|  | (865) | (865) | (864) | (864) | (864) | (865) | (865) | (865) | (865) | (864) |
| **Mental illness** | .353** | .301** | .229** | .193** | .291** | .342** | 1 | .355** | .289** | .187** |
|  | (865) | (865) | (864) | (864) | (864) | (865) | (865) | (865) | (865) | (864) |
| **Alcohol abuse** | .441** | .361** | .129** | .231** | .306** | .439** | .355** | 1 | .368** | .206** |
|  | (865) | (865) | (864) | (864) | (864) | (865) | (865) | (865) | (865) | (864) |
| **Drug abuse** | .298** | .238** | .115** | .277** | .182** | .378** | .289** | .368** | 1 | .371** |
|  | (865) | (865) | (864) | (864) | (864) | (865) | (865) | (865) | (865) | (864) |
| **Incarceration** | .277** | .186** | .181** | 0.051 | .169** | .271** | .187** | .206** | .371** | 1 |
|  | (864) | (864) | (863) | (863) | (863) | (864) | (864) | (864) | (864) | (864) |

**Correlation is significant at the 0.01 level (2-tailed); *Correlation is significant at the 0.05 level (2-tailed).
