# Supplementary material for: Maternal adverse childhood experiences and their association with preterm birth: secondary analysis of data from universal health visiting
Source: BMC Pregnancy Childbirth. 2022 Feb 16;22:129. doi: 10.1186/s12884-022-04454-z (PMC8848970; doi:10.1186/s12884-022-04454-z)
Supplement: Supplementary file 3 — Additional file 3: Table A3. Logistic regression of ACE exposure, demographics and their association with preterm birth among healthy* mothers. [file 12884_2022_4454_MOESM3_ESM.docx]

**Table A3. Logistic regression of ACE exposure, demographics and their association with preterm birth among healthy* mothers**

|  |  | **Preterm birth** | | | |
| --- | --- | --- | --- | --- | --- |
|  |  | **AOR** | **Low CI** | **High CI** | **p** |
| **Total ACE exposure** | 0 ACEs | (ref) |  |  | 0.023 |
|  | 1 ACE | 0.60 | 0.19 | 1.86 | 0.374 |
|  | 2-3 ACEs | 1.04 | 0.33 | 3.31 | 0.944 |
|  | ≥4 ACEs | **3.89** | **1.40** | **10.80** | **0.009** |
| **Age category (years)** | 16-25 | (ref) |  |  | **0.043** |
|  | 26-35 | 2.08 | 0.64 | 6.72 | 0.222 |
|  | >36 | **5.44** | **1.35** | **21.89** | **0.017** |
| **Ethnicity** | Other | 0.27 | 0.04 | 2.09 | 0.210 |
| **Pilot area** | Swansea | (ref) |  |  | 0.870 |
|  | Carmarthenshire | 0.78 | 0.31 | 2.00 | 0.609 |
|  | Blaenau Gwent | 0.99 | 0.36 | 2.68 | 0.980 |
| **Deprivation** | Flying Start | 0.97 | 0.39 | 2.43 | 0.953 |
| **First child** | Yes | 1.44 | 0.66 | 3.15 | 0.364 |
| **Relationship status** | Single | (ref) |  |  | 0.709 |
|  | Partnered/cohabiting | 0.75 | 0.15 | 3.66 | 0.722 |
|  | Married | 0.57 | 0.11 | 3.09 | 0.518 |
|  | Not disclosed | 0.43 | 0.07 | 2.49 | 0.344 |

*Mothers with no known chronic health conditions.
